# Investigation of dermal exposure to heavy metals (Cu, Zn, Ni, Al, Fe and Pb) in traditional batik industry workers

**DOI:** 10.1016/j.heliyon.2022.e08914

**Published:** 2022-02-09

**Authors:** Katharina Oginawati, Suharyanto, Septian Hadi Susetyo, Gintang Sulung, Muhayatun, Nurul Chazanah, Siska Widya Dewi Kusumah, Nurul Fahimah

**Affiliations:** a Study Program of Environmental Engineering, Faculty of Civil and Environmental Engineering, Institut Teknologi Bandung, Indonesia; b National Nuclear Energy Agency of Indonesia, Indonesia

**Keywords:** Occupational health, Batik industry, Heavy metals, Dermal exposure, Hazard quotient, Hazard index

## Abstract

Batik is an Indonesian cultural heritage that has been designated by UNESCO. Batik industry is one of the industries that applies the synthetic dyes, beside natural ones which have long been used in producing Batik particularly in the modern production. The purpose of this research investigated heavy metals on workers in batik industry, which focuses on dermal detection as portal of entry. Samples of dermal intake of workers were examined with *cross-sectional approach*, while non-worker samples of resident living surrounding the industry were used as control. *Dermal intake* on workers and non-workers were examined using *Patch Filter*. Heavy metals content of the synthetic dyes used in the batik production and those in the patch filter which was attached to worker's skin during sampling period were analyzed using X-Ray Fluorescence (XRF) method. The XRF measurement result of the synthetic dyes shows a detection of several heavy metals including Cu and Zn as the highest detected concentration, while the XRF measurement of the patch filter detects several heavy metal contents, which include Cu, Zn, Ni, Al, Fe and Pb. The highest detected heavy metal concentration found in the patch filter were Cu, Zn and Ni. Meanwhile, the highest detected heavy metal concentration of Pb was found in workers in the stamping process. The result indicates that highest Hazard Quotient (HQ) values for Cu, Zn, Mn, and Fe were found in workers of dyeing process compared to those in other processing stages.

## 1 Introduction

Batik has long been attributed to the Indonesian heritage and has been globally recognized –officially declared by UNESCO in 2009. It has been important entity relating to the development of various sectors particularly tourism sector. Several cities in Indonesia have been well-known with batik products such as Cirebon, Pekalongan, Yogyakarta and Surakarta representing various local cultures where it is originated. In one of the centres of batik industry in the area of Yogyakarta, there are 108 batik industries in one of its regency (Kulon Progo Regency/District) in 2015 [1], in which industry is the most dominant and among a common type in the batik production.

Batik industry is apparently one of the industries being more capable to cope with external environment change. This could be impelled by the characterstic of entrepreneur driven and not by the market driven [2]. According to report issued by the Ministry of Trade, there are about 50 thousand batik industries in Indonesia with approximately 800 thousand people in small-medium enterprise and about 5 thousand people in big industry are employed [3, 4].

There are various types of batik: written batik, stamped batik, a combination written-stamped batik, printed batik, and woven batik. As regard the motive, batik is classified into rural batik such as that from Central Java and coastal batik such as that from cities in the northern coastal of Java Island [5]. In the written and combined written-stamped batik production, a special tool called *canting*, which is a pen-like instrument, is used to draw batik pattern with a melted wax applied onto the raw fabric. Stamped batik is similar to written batik in terms of the use of dye-resisting wax, except the pattern applied onto the cloth is by using a stamp instead of pen-like canting. The use of stamp shortens the production time compared to the latter method.

Natural coloring have been traditionally used in batik production. It also contains substances that are more biodegradable and less toxic [6]. However, the biggest challenge in meeting natural dyes is the duration of the process because the processing time for synthetic dyes is shorter than natural dyes [7, 36]. Boiling textiles with chemicals can increase penetration and sharpen colors [26].

Along with its wide recognition and consequent production, exposure of heavy metals from batik production on the workers could be a concern due to the use of synthetic dyes. Heavy metal content in the synthetic dyes is potentially found in textile production. Some reactive dyes contain heavy metals such as Cd, Cu and Pb [8]. Various dyes used in textile industry also contain heavy metals such as Cu, Cr, Cd, Fe, Pb, Ni, and Zn [9, 10].

On the other hand, with regard to the potential risk of exposure, batik industry have a potential risk of heavy metal exposure through various portal of entry including through skin (dermal) [11], inhalation, and oral [12]. Heavy metal - containing dyes were detected in Batik wastewater and also in workers exposure [13]. Health impact of heavy metal exposure has been revealed by various studies including effect on nervous system [14]. Heavy metals can affect the central nervous system leading to mental disorders, damage blood constituents, damage lungs, liver, kidneys and other vital organs [15]. In the long term in the body with repeated contact, heavy metals can damage nucleic acids, cause mutations, mimic hormones so that they can interfere with the endocrine and reproductive systems and eventually can cause cancer [27].

Furthermore, dermal exposure to heavy metal of industry workers using dyestuff has been also reported including detected Cd, Cr, Ni, and Pb on the hands of workers' skin [16]. Heavy metal content in dyestuff similar to that used in textile production has been reported including through the detected heavy metals in dyeing wastewater [9]. The risk of exposure is aggravated by the condition in which most of batik industry workers do not wear personal protective equipment (PPE). Only about 13.7% of workers in the study area wear PPE [7]. With this, it is highly important that exposure of batik workers to heavy metals be investigated. Referred to those studies, potential risk of heavy metal exposure on textile and batik industry should be highly considered. Accordingly, this study examines one of the potential risks i.e. skin exposure through studying the possible dermal adverse effect of the heavy metals on batik industry workers. This issue needs to be investigated in order to prevent growing health impact of the workers could have reached a level, which needs high attention from the health and safety point of view. This study aims to identify possible heavy metals containing substance used in batik industry and to analyze the risk associated with dermal exposure of the workers. Assessment of risk with regard to reference limit of heavy metal exposure on the workers was subsequently conducted.

## 2 Material and method

### 2.1 Study area

**Figure 1:**
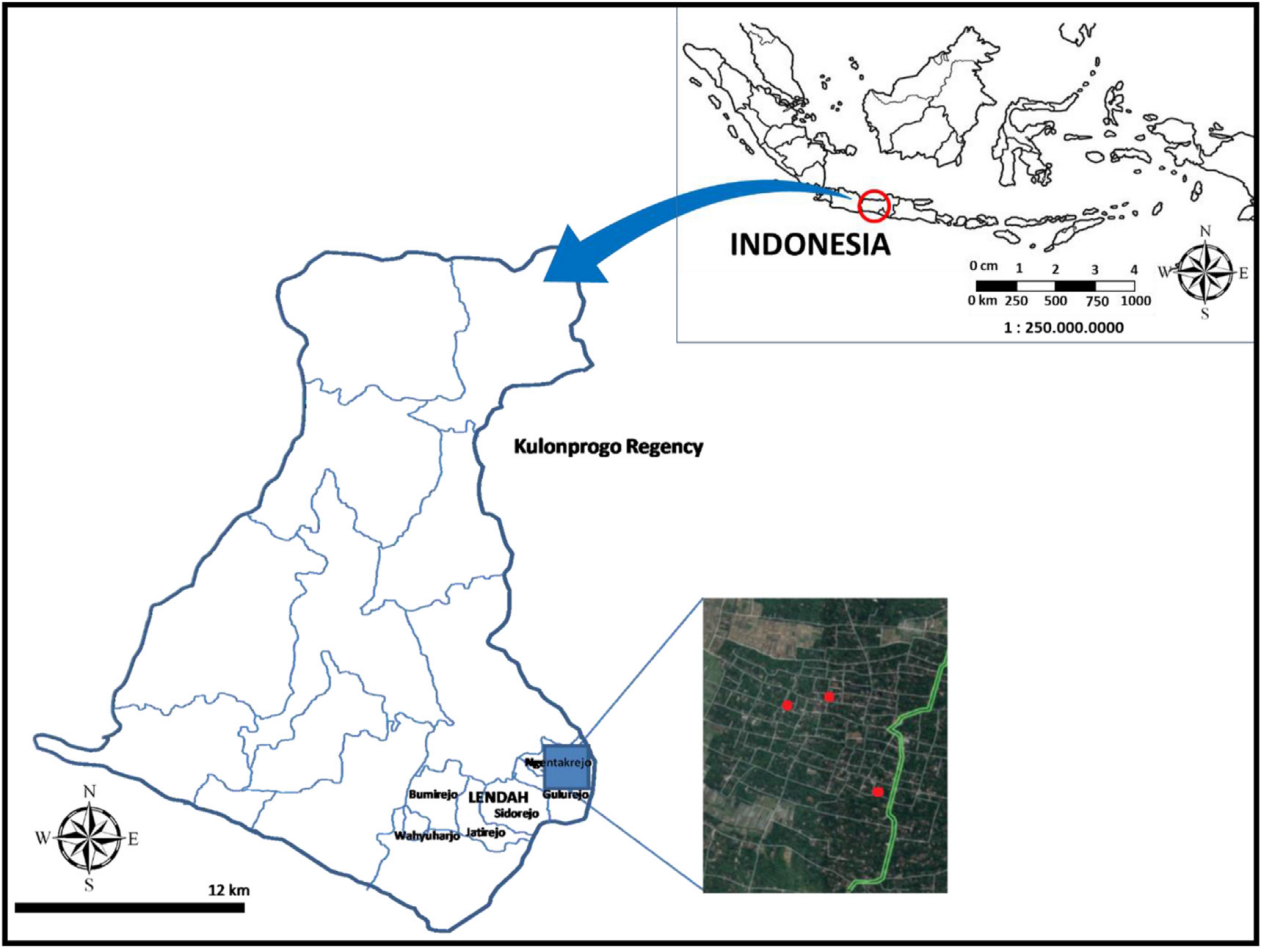
Study Area of three batik industries in Lendah District, Kulon Progo, Yogyakarta Province, Indonesia.

The study was conducted in three batik industries in two villages (Ngentakrejo and Gulurejo) located at district of Lendah, Kulon Progo about 40 km away from The City of Yogyakarta, which is one of the centers for batik industry. Lendah District is one of 12 Districts of Kulon Progo Regency in Yogyakarta Province. The Regency of Kulon Progo is located between 7^o^38′42'' - 7^o^59′3″ South Latitude and 110^o^1′37'' - 110^o^16′26″ East Longitude with a total area of 58,627.5 Ha, it is displayed in Figure 1 [17].

### 2.2 Batik production stages

**Figure 2:**
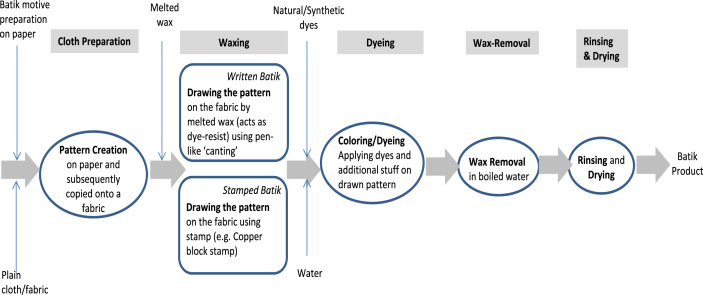
Production stages for written/stamped batik.

In general, batik production stages for written and stamped batik consist of (i) cloth preparation and pattern creation, (ii) waxing, (iii) dyeing, (iv) wax removal, (v) rinsing and drying. In the cloth preparation, batik motive is firstly prepared on paper and is subsequently copied onto the plain cloth/fabric from which the batik pattern is created. Next, in the waxing stage, melted wax is applied to draw the pattern using pen-like *canting* for the written batik and using a stamp for stamped batik by which the wax covers the fabric and prevents the dyes from coloring the cloth and keeps the original color of the fabric. Multiple coloring processes may apply to obtain various color combination with that similar step. Coloring/Dyeing stage follows the next stage in which dyestuff using natural or synthetic dyes is applied. Some other substances might be added in this stage to gain better result of this coloring stage. Wax removal using boiled water and followed by rinsing and drying are the subsequent stages to retain the final batik product. Production stage are able shown in Figure 2.

The resist-dye wax is essential material used in the batik production owing to its essential role in handling uncontrolled all-direction spread of the dye on the cloth. Additionally the wax is also used in order to obtain a clear-cut colorfast appearance of the batik product [18]. The use of natural and also synthetic dyes has been applied in the batik production. This includes native plants for natural brown/yellow/red colors and also indigo for synthetic dark blue color as an example of traditional colors used for batik in Central Java [18].

Although the batik producers in some small and medium enterprices (SMEs) tend to return to use natural coloring such as mango leave and bark of mahoni [5], the use of synthetic dyes is still widely used due to less expensive selling price of the product compared to that with natural coloring. As a comparison, the price of batik product with synthetic dyes costs IDR 125,000 (1 USD is about IDR 14.000), which is lower than that of natural coloring that costs IDR 300,000 to 500,000 [5]. Therefore, related to this study, a concern over heavy metals content, which are among constituents contained in the batik dyeing process as well as the consequent batik wastewater involving the use of synthetic coloring [19] becomes a focus of this research.

### 2.3 Health risk analysis

**Figure 3:**
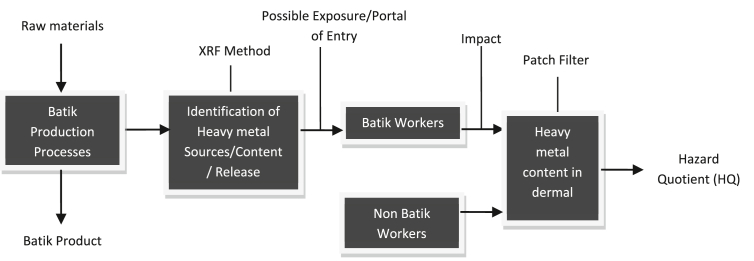
Research framework.

As part of hazard identification and evaluation, the study attempts to identify possible hazard of heavy metal exposure from batik production and analyze potential health risk for the workers. Dermal exposure concentration was approached by determining exposure duration and frequency on the exposed area of workers' hand with patch filter attached. The use of patch filter was directed towards an estimation of potential and applied doses. Research framework is able shown in Figure 3.

The conducted study applied cross-sectional approach with two groups of respondents with field observation, questionnaire, dermal exposure measurement were conducted to 30 workers of batik industry in three area in Lendah District, and to 30 non-production workers and surrounding residents as control. Purposive sampling was undertaken by limiting the samples of workers based on (i) age of 18 or older; (ii) minimum three-month work experience in canting, stamping, dyeing process of batik production; (iii) not currently working with possible heavy metals exposure such as wielding or other heavy metals works; (iv) in healthy condition and not consuming any drugs. While criteria for control were set as follows: (i) non-worker in batik industry; (ii) not working at the production stages e.g. marketing division or administrative workers.

Minimum number of samples referred to Occupational Exposure Sampling Strategy Manual, NIOSH [20]. Data of respondents regarding age, gender, work experience, work duration, the use of personal protective equipment, disease record, life style and habit including smoking, alcoholic drink consumption, and symptoms of heavy metal exposure were also collected.

**Figure 4:**
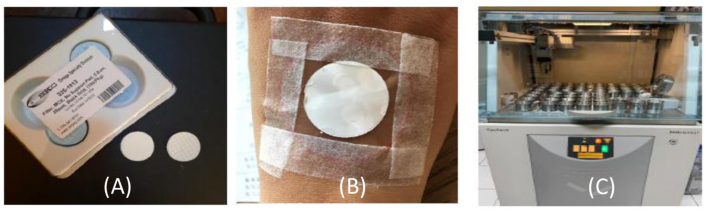
(A) MCE filter used in the research, (B) applied on worker's hand, (C) Filter and XRF Instrument.

Dermal exposure measurement was conducted using Mixed Cellulose Esther (MCE) filter with a diameter of 25 mm and the pore size of 0.8 um. The filter was attached on the hand of workers and non-workers for 3 h. Subsequently, the filters were examined to detect the presence of heavy metals using X-Rays Fluorescence (XRF) Method. The investigation of dermal exposure for workers in production stages comprise: (i) canting (pattern drawing) division, (ii) stamping division, (iii) dyeing division. The result of measurement was then compared to the reference dose (R_f_D) released by the US-EPA for assessing the HQ. Heavy metal content of raw material used in the batik production of dyeing stage was also analyzed using X-Ray Fluorescence (XRF). The tools are able shown in Figure 4.

Average Daily Dose (ADD) was determined after measurement result of heavy metal content in the filter, which is calculated as follows:

(1) $$ADD=\;\frac{\left(DA\;x\;EV\;x\;ED\;x\;EF\;x\;SA\right)}{\left(BW\;x\;AT\right)}$$

where ADD (mg/kg.day), DA is heavy metal dose (mg/cm^2^.event), EV is exposure frequency (event/day), ED is exposure duration (years), EF is exposure frequency (hours/year), SA is available skin surface area for contact (10,700 cm^2^), BW is Body Weight (kg), and AT is average time (days) [21, 22].

In evaluating the risk of batik industry workers, HQ Index was determined comparing the calculated ADD value to the R_f_D [Eq.2]. The toxic effect of the heavy metal exposure will be a concern when the HQ Index is more than 1 (HQ > 1) [23,24,25].

(2) $$HQ=\frac{ADD}{RfD}$$

(3) HI=∑HQ

### 2.4 Ethical approval

The ethical clearance was conducted closely between the parties involved in the research. The two-way agreement was obtained by verbal communication and signed a handwritten agreement. The Ethics Committee has approved this research of Padjajaran University, Bandung, Indonesia with No: 353/UN6.KEP/EC/2020.

## 3 Result and discussion

### 3.1 Results of measurement

**Figure 5:**
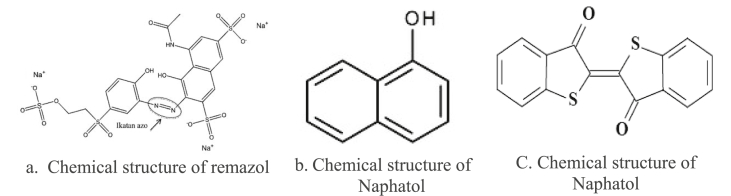
Chemical structure of Remazol, naphthol, and indigosol

**Figure 6:**
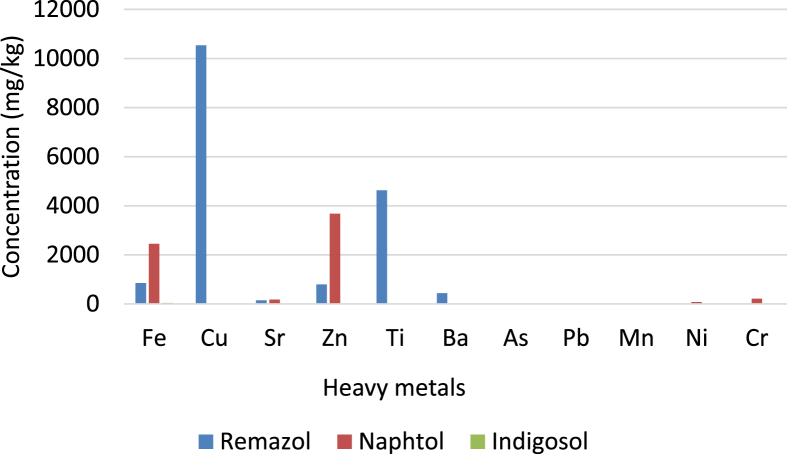
Heavy metal concentration in the synthetic dyes used in batik industry observed in the study and heavy metal concentration in three types of synthetic dyes used in batik industry.

Based on the observation during the study, the dominant synthetic dyes used in the batik production in the study area were remazol, naphthol, and indigosol. The Chemical structure of Remazol, naphthol, and indigosol shown in Figure 5. The result of XRF measurement of the those synthetic dyes used shows a detection of several heavy metals including Cu, Ti, Zn and Fe, which were among the highest concentration detected. It is displayed in Figure 6. Those detected heavy metals contained in the synthetic dyes could become a potential source of exposure, particularly to workers in the dyeing and other processes, in which metal fume could possibly be generated.

**Figure 7:**
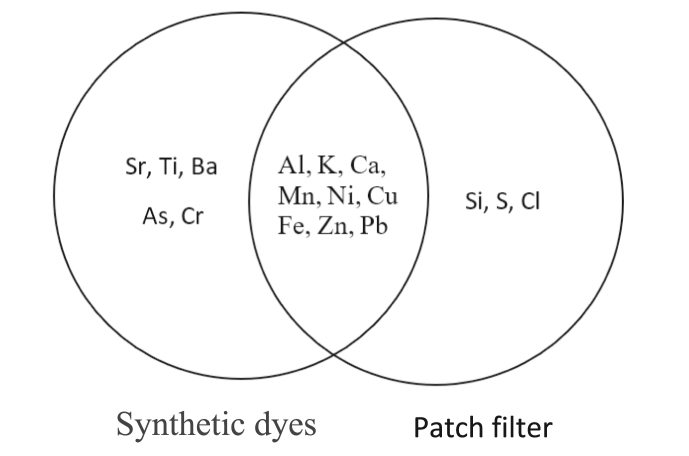
Heavy metal contents both in the synthetic dyes and in the patch filter.

The XRF measurement of the patch filter detects several heavy metal contents including Al, Ni, Cu, Zn, and Pb. It is able seen in Figure 7. Those heavy metal contents were similar to those found in the used synthetic dyes. This indicates that those metals could have possible exposed to the batik industry to a certain level that would be of a concern.

The production of batik in the study area operates 8 h a day and six days a week. Based on observation and interview with respondents, about 56.5% of workers have more than five years work experience in batik industry, while 27% of those have 1–5 years work experience and remaining 16.5% of those have less than one year experience. Regarding workers with PPE during production stage, only a minority of workers (13.7%) wear the PPE. The remaining majority did not wear PPE with inconvenience is among the major reason.

The highest heavy metal concentration found in the patch filter with associated production stages can be seen in Table 1. The highest detected heavy metal concentration of Cu, Ni, Zn and Fe in the patch filter, which is 9.29, 2.11, 102.13 and 132.60 ng/cm^2^, respectively were found in workers of dyeing process. Meanwhile, the highest concentration of Pb was found in workers of stamping process (6.91 ng/cm^2^). The pattern each heavy metals exposure have different especially in Pb, it was caused in the canting, stamping and dyeing processes, different liquid materials were used so that they have different concentrations for each exposure to heavy metals. All heavy metals detected, the dyeing process is a part of the work that has a higher level of exposure to heavy metals than other processes. This result is because in the dyeing section, workers were in direct contact with dye solutions containing heavy metals. Heavy metals can enter the body by absorption through the skin and can be a potential danger to human health [28]. However, the amount absorbed varies greatly, depending on the chemical form of the metal and the age and nutritional status of the individual [29]. In addition, it is also influenced by the time and dose of exposure [30].

**Table 1:** Dermal heavy metal contents including in the patch filter.

Name	Concentration (ng/cm^2^)					
	Mn	Fe	Ni	Cu	Zn	Pb
*Canting* Process	4.74	47.37	2.09	8.52	9.41	5.88
Stamping Process	3.46	29.50	1.84	0.83	16.28	6.91
Dyeing Process	6.78	132.60	2.11	9.29	102.13	6.79

### 3.2 Dermal intake estimate

To evaluate the risk of heavy metals exposure of the workers, ADD for each worker was determined. The data is displayed in Table 2. The frequency of exposure was based on the work duration of 8 h per day, six days per week. The result of AD was shown in Table 2. The highest ADD of heavy metals differs for each production stages. The highest ADD for Zn and Pb occurs for workers in stamping stage with 743.25 and 301.59 ng/kg-day, furthermore Mn and Ni occur for worker in canting process with 206.03 and 8.38 ng/kg-day, while Fe and Cu occurs for workers in the dyeing process with 2476.79 and 277.29 ng/kg-day.

**Table 2:** ADD values of heavy metals in each production stage.

Kinds process		ADD (ng/kg-day)					
		Mn	Fe	Ni	Cu	Zn	Pb
*Canting* Process	Min	0.00	47.24	0.23	0.00	35.35	127.65
	Max	764.98	7681.66	29.82	1078.24	1384.23	487.47
	Average	206.03	2220.77	8.38	197.10	414.52	264.16
Stamping Process	Min	79.27	186.21	0.14	0.00	217.88	181.22
	Max	293.15	2788.24	2.35	44.73	2638.36	441.68
	Average	152.70	1271.05	1.26	14.40	743.25	301.59
Dyeing Process	Min	0.00	0.00	0.49	0.00	12.37	134.24
	Max	538.99	5793.94	10.25	1880.85	2340.52	465.75
	Average	171.37	2476.79	4.56	277.29	698.21	280.45

### 3.3 Hazard quotient (HQ) estimate

To evaluate whether or not the hazard level of heavy metal exposure (and intake) to batik industry workers in the study area has occurred, HQ index was calculated for each heavy metal. Furthermore, the result of this HQ calculation can be used to evaluate an overall hazard level for all heavy metals detected, which is expressed in total HQ, if fthe value of higher than 1 indicates a potential health hazard to workers. RfD for each heavy metal was used for HI calculation. The result shows that HQ values of workers in the dyeing process for Mn, Fe, Cu, and Zn are the highest compared to other processing stages. The HQ values are shown in Table 3.

**Table 3:** HQ values related to heavy metal dermal exposure of batik industry workers in the study area.

Heavy metal	HQ		
	*Canting* Process	Stamping Process	Dyeing Process
**Mn**	70.01471	0.01091	0.01473
**Fe**	0.00317	0.00182	0.00636
**Ni**	0.00042	0.00006	0.00025
**Cu**	0.00493	0.00036	0.00686
**Zn**	0.00138	0.00248	0.01144
**Pb**	0.07547	0.08617	0.08000
**Total HQ**	0.10009	0.10179	0.11964

Judging from the HI value, workers in the dyeing section have the highest HI value when compared to workers in the canting and tasting sections. This result is presumably because the dyeing part is in direct contact with the heavy metals in the dyestuff. Direct contact occurs when workers carry out the dyeing process. The results of this study are also supported by other studies that heavy metals can be present in the dyeing process in the textile industry and toxic and dangerous heavy metals can accumulate in the human body [31, 32].

While the results of the calculation of the value of the HI for the three industries show results that are not much different between the three industries. The highest HI value is found in the SAB industry with worker conditions dominated by workers in the dyeing section and narrow workspaces and workers who do not use personal protective equipment. SAB industry is the company that focus in the production of textile. The workers in the dyeing department have a higher potential for exposure to heavy metals through the skin compared to other work processes such as canting and stamping, in addition to poor working environmental conditions such as closed workspaces and the unavailability of personal protective equipment can contribute to the risks that exist in the industry of SAB batik. Other studies have found a relationship between work position and use of personal protective equipment with levels of heavy metals in the body and concluded that workers who wore masks and/or cotton gloves had significantly lower blood levels of Cd and Pb than those who did not [33]. In addition, the presence of ventilation allows workers to carry out work without risk to health [34].

Overall, the HI value for dermal exposure by batik workers has not exceeded the value 1. It means that the exposure has not caused any adverse effects for workers. However, the HI value in this study did not indicate a long-term and comprehensive risk, meaning that heavy metal exposure does not only expose workers through the skin route, workers can be exposed to heavy metals through other routes such as inhalation or ingestion. The occupational risk stems from different exposure pathways for heavy metals namely through hand-mouth ingestion, dermal absorption and air inhalation, where the HI of hand-mouth ingestion is 5–6 and 2–3 orders of magnitude higher than skin absorption and air inhalation respectively [35]. If exposure occurs over a long period of time, heavy metal compounds can bioaccumulate in the body and can cause adverse health effects.

## 4 Conclusion

The study showed that a detection of several heavy metals including Cu and Zn as the highest detected concentration in synthetic dyes used in batik industry while several heavy metals were also detected in the patch filter attached on the workers' skin, which include Cu, Zn, Ni, Al, and Pb. Those heavy metal contents on the patch filter were similar to those found in the synthetic dyes. This indicates that those metals may possibly expose the workers at a certain level. The detected concentrations for Cu, Zn, and Ni in the patch filter were 9.29, 102.13, and 2.11, ng/cm^2^, respectively that were attached to workers’ skin of dyeing process. Meanwhile, the detected heavy metal concentration of Pb of 6.91 ng/cm^2^ was found in workers of the stamping process. The result indicates that highest HQ values for Cu, and Zn, Mn, and Fe were found in workers of dyeing process compared to those in other processing stages. However, those indexes were still below the limit (HI < 1) and do not yet express a concern over health adverse effect. Nevertheless, further studies are required to investigate other routes of heavy metal exposure of the 0batik industry workers including inhalation and oral portal of entries for obtaining overall risk estimate.

## Ethical approval and consent to participate

The author's statement of ethical clearance was conducted closely between the parties involved in the research. The two-way agreement was obtained by verbal communication and signed a handwritten agreement. The Ethics Committee has approved this research of Padjajaran University, Bandung, Indonesia with No: 353/UN6.KEP/EC/2020.

## Declarations

### Author contribution statement

Katharina Oginawati: Conceived and designed the experiments; Performed the experiments.

Nurul Chazanah: Conceived and designed the experiments; Wrote the paper.

Suharyanto: Performed the experiments; Wrote the paper.

Gintang Sulung & Septian Hadi Susetyo: Performed the experiments; Analyzed and interpreted the data; Wrote the paper.

Siska Widya Dewi Kusumah & Nurul Fahimah: Analyzed and interpreted the data; Wrote the paper.

Muhayatun: Contributed reagents, materials, analysis tools or data; Wrote the paper.

### Funding statement

This work was supported by P3MI ITB 2020 and Riset Kolaborasi Indonesia (RKI).

### Data availability statement

Data included in article/supplementary material/referenced in article.

### Declaration of interests statement

The authors declare no conflict of interest.

### Additional information

No additional information is available for this paper.
